# Adding DNA barcoding to stream monitoring protocols – What’s the additional value and congruence between morphological and molecular identification approaches?

**DOI:** 10.1371/journal.pone.0244598

**Published:** 2021-01-04

**Authors:** Simone Behrens-Chapuis, Fabian Herder, Matthias F. Geiger

**Affiliations:** Zoologisches Forschungsmuseum Alexander Koenig, Bonn, Germany; Universita degli Studi di Roma La Sapienza, ITALY

## Abstract

Although aquatic macroinvertebrates and freshwater fishes are important indicators for freshwater quality assessments, the morphological identification to species-level is often impossible and thus especially in many invertebrate taxa not mandatory during Water Framework Directive monitoring, a pragmatism that potentially leads to information loss. Here, we focus on the freshwater fauna of the River Sieg (Germany) to test congruence and additional value in taxa detection and taxonomic resolution of DNA barcoding vs. morphology-based identification in monitoring routines. Prior generated morphological identifications of juvenile fishes and aquatic macroinvertebrates were directly compared to species assignments using the identification engine of the Barcode of Life Data System. In 18% of the invertebrates morphology allowed only assignments to higher systematic entities, but DNA barcoding lead to species-level assignment. Dissimilarities between the two approaches occurred in 7% of the invertebrates and in 1% of the fishes. The 18 fish species were assigned to 20 molecular barcode index numbers, the 104 aquatic invertebrate taxa to 113 molecular entities. Although the cost-benefit analysis of both methods showed that DNA barcoding is still more expensive (5.30–8.60€ per sample) and time consuming (12.5h), the results emphasize the potential to increase taxonomic resolution and gain a more complete profile of biodiversity, especially in invertebrates. The provided reference DNA barcodes help building the foundation for metabarcoding approaches, which provide faster sample processing and more cost-efficient ecological status determination.

## Introduction

Species richness in freshwater ecosystems is increasingly endangered by the consequences of climate change, environmental pollution, overexploitation, river fragmentation or flow regulation, and invasive species [1–3]. Therefore, protection of aquatic habitats and their functions, combined with prevention of further deterioration and initiation of restoration, has become an important task in Europe and elsewhere.

The international Convention on Biological Diversity [4] defined a general framework for counteracting degradation through restoration and management of aquatic ecosystems, followed by national and regional conservation strategies and action plans. The resulting programs–implemented for example in the USA (the National Aquatic Resources Survey (NARS; previously known as EMAP)), in Canada (the Canadian Aquatic Biomonitoring Network (CABIN)), in South Africa (the National Aquatic Ecosystem Health Monitoring Program (NAEHMP)) or in Australia (the AUStralian RIVer Assessment System (AUSRIVAS))–have all in common that they aim to acquire detailed data that describe the ecological health and trends of freshwater bodies, ideally based on continuous monitoring of aquatic indicator taxa [5].

In the European Union the required aquatic quality assessment became legally binding through the Water Framework Directive [6], which aims to restore in all member states a ‘good ecological status’ of each surface waterbody at the latest by 2027. This directive changed the focus of water management from simple pollution control to measuring aquatic ecosystem integrity and health [7], by using five “biological quality elements” (BQEs): fishes, aquatic macroinvertebrates, phytoplankton, macroalgae, and macrophytes, supplemented by chemical and hydromorphological quality indicators (see annex II and V). The distance between the observed conditions to defined undisturbed reference water bodies (i.e. water bodies with unaltered type-specific water quality and morphology, inhabited by taxa expected in the absence of human pressure–Directive 2000/60/EC; see for NRW www.lanuv.nrw.de/fileadmin/lanuvpubl/0_lua/merk29web_kl.pdf, https://www.flussgebiete.nrw.de/fischgewaessertypen-5585) is then calculated as the Ecological Quality Ratio (EQR), and finally translated into the quality categories: high, good, moderate, poor, or bad. A categorization less than good always requires action and management to improve site conditions until a good ecological status is reached.

In the current WFD monitoring protocols, the identification of BQEs is based on morphological identification and counting [8], making the accuracy and level of taxonomic resolution achieved always dependent on the individual knowledge and experience of the respective investigator (often just a single person or a small team per BQE), while professional taxonomists are getting rare even among biologists [9,10]. Besides the overall potential for misidentifications [8,11–13], particularly the morphological classification of early fish life stages and immature aquatic invertebrates with insufficient diagnostic characters is challenging, time consuming, and therefore considered to be costly [14–17]. This results in severe problems in species determination, with cryptic species or lineages remaining undetected [17–19]. Hence, such “problematic” organisms are usually identified only to coarser taxonomic levels, i.e. to genus, family or order, or are even excluded [13,17]. Information based on higher-level taxonomy can be sufficient in standard bioassessments [20,21], but valuable information about species-specific ecological requirements and stressor tolerances may remain unnoticed [19,22]. This may in turn lead to potentially inaccurate water quality assessments and mismanagement of freshwater ecosystems [23,24].

From a scientific point of view, solutions like DNA-based techniques appear promising to overcome these shortcomings [e.g. 24–28]. Recent studies showed that in particular DNA barcoding using a short sequence (~ 658 bp) of the mitochondrial cytochrome oxidase subunit I (COI) [29,30] enables a fast and reliable taxon identification to species-level of whole or even parts of specimens across any life stage which already offers great promise in advancing freshwater bioassessment and monitoring routines [13,24,25].

As part of the German Barcode of Life initiative (www.bolgermany.de) we conducted an applied study using DNA barcoding and classical approaches on the faunal quality elements of the Sieg, a river with a catchment area of approx. 2900 km^2^. The Sieg enters the Rhine close to Bonn in western Germany and is classified according to the German stream typology [31,32] as a type 9.2 ‘large highland river’. Such rivers are typically characterized by highly diverse habitat structures and aquatic animal communities [32,33], revealing a suitable model system for exploring the potential of DNA barcoding in monitoring routines. Hence, we exemplary use river type-specific fish and macroinvertebrate assemblages of the Sieg to evaluate the performance of both methods: We directly compare identification congruence and taxonomic resolution, and provide an authentic estimation for cost and time effort. We also deliver additional reference DNA barcodes for German freshwater fishes and macroinvertebrates, evaluated through BOLD’s Barcode Index Number (BIN) assignment.

## Materials and methods

### Sampling

Ethic statement: All applicable international, national, and/or institutional guidelines for the care and use of animals were followed. Permissions were obtained beforehand from the responsible German authorities: Amt für Natur- und Landschaftsschutz, Bauvorhaben, Landschaftsplanung, Artenschutz (exemption from the prohibitions of the Bundesnaturschutzgesetzes in line with § 45 Abs. 7 Nr. 3, § 44 Abs. 1 Nr.1 and § 67 Abs.1 - in combination with the Landschaftspläne 6, 7, 9, 10 und 15 sowie der ordnungsbehördlichen Verordnung über das Naturschutzgebiet und Landschaftsschutzgebiet, Siegaue), Bezirksregierung Köln (sampling permission: § 4 Abs. 3 LFischVO; Az. 51.3–1.7.9-187/12) and Rechts- und Ordungsamt–untere Fischereibehörde (permission for electro-fishing following § 10 Abs. 1 Ziffer 1 SGV.NRW 793).

Sampling campaigns were conducted in the years 2012 to 2014, focussing on aquatic macroinvertebrate species and the different developmental stages of fishes. Sampling was performed at the River Sieg in North Rhine-Westphalia (NRW) by two WFD monitoring and quality assessment experts for the respective BQEs, following standardized field protocols used in German WFD stream monitoring routines (aquatic invertebrates: [34,35]; fishes: [36]).

**Fig 1:**
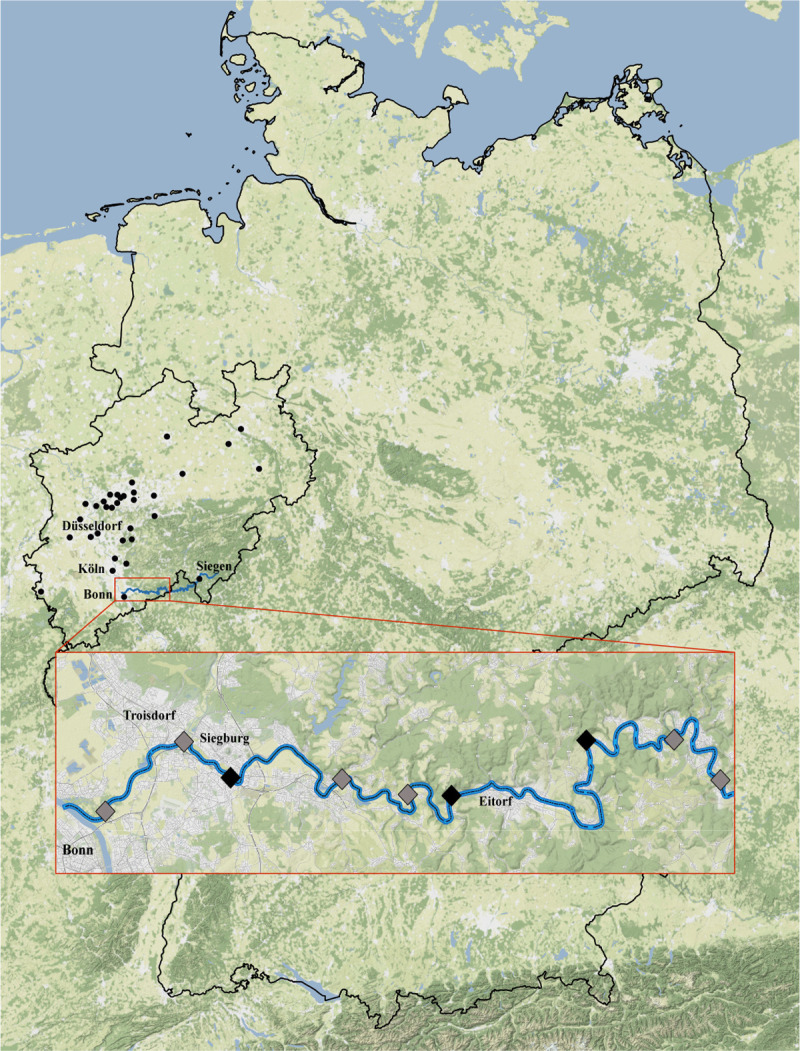
Nine main sample locations of fishes at the River Sieg, Germany. From left to right: Bergheim, Aggermündung, Pleisbachmündung, Brölmündung, Bülgenauel, Happach, Röcklingen, Schladern, Irsenbachmündung. The six points (Bergheim, Aggermündung, Brölmündung, Bülgenauel, Schladern, Irsenbachmündung) where additionally macroinvertebrates were sampled are marked in grey (Map tiles by Stamen Design).

Macroinvertebrates were collected and then morphologically identified by the limnologist Dr. Guido Haas (www.hbio-hessen.de), who regularly implements the required WFD monitoring and quality assessment for the BQE ‘aquatic macroinvertebrates’ by order of the NRW state government. The specimens were sampled at six main sample locations (grey, Fig 1) using the standardized multihabitat sampling technique described by Meier et al. ([34,35]–a modified version of AQEM/Star method). Following this approach different microhabitats present are sampled proportional to their coverage at each sample site. Each substrate type (Mega-, Makro-, Meso-, Mikrolithal, Akal, Psammal-/pelal, Argyllal, Xylal, Technolithal 1, CPOM, submerse Makrophyten, Algen, lebende Teile terr. Pflz.) with at least 5% cover is sampled by kick-net sampling and manual searching using a hand net with a 0.25x0.25 m frame (mesh-size 0.5 mm; depth of 70 cm), resulting in 20 ‘sampling units’ and a total river bottom sampling area of 1.25m^2^ per monitoring site; rare microhabitats (cover <5%) were considered by including them in one additional (no. 21) sampling unit. Invertebrate samples were processed by ‘live-sorting’ in the field (see [34,35]) and the required number of representatives from each taxon (excluding colony-forming taxa) taken for detailed identification in the laboratory and subsequent DNA barcoding routines; all remaining individuals were returned alive (see [34,35]). Additional morphologically identified macroinvertebrate samples from two small tributaries to the river Sieg were included to increase taxa diversity for the comparative analysis: one part from the Wahlbach (Table 1), and one from the Krabach, provided by the INRES (Institut für Nutzpflanzenwissenschaften und Ressourcenschutz) institute. Invertebrate taxa numbers were counted in accordance to the field protocol, i.e., *Ecdyonurus* sp. (5053 taxa ID number—see [37]: national operational German taxa list) and *Ecdyonurus insignis* (5046 taxa ID number) were counted as 2 taxa. The aquatic invertebrate samples are disposed in the GBOL collection of the Zoologisches Forschungsmuseum Alexander Koenig (ZFMK) in Bonn.

**Table 1:** Sample locations of fishes and aquatic invertebrates in the River Sieg and its tributaries.

				FiGT	organisms
	sample point	latitude	longitude	Fish water type	sampled
**1**	**Sieg–Bergheim**	50.7657	7.1074	11	fishes / invertebrates
**2**	**Sieg–Aggermündung**	50.8004	7.1739	11	fishes / invertebrates
**3**	**Sieg–Pleisbachmündung**	50.7826	7.2133	11	fishes
**4**	**Sieg–Brölmündung**	50.7818	7.3076	10	fishes / invertebrates
**5**	**Sieg–Bülgenauel**	50.7741	7.3631	10	fishes / invertebrates
**6**	**Sieg–Happach**	50.7736	7.4000	10	fishes
**7**	**Sieg–Röcklingen**	50.8011	7.5140	9	fishes
**8**	**Sieg–Schladern**	50.8011	7.5882	9	fishes / invertebrates
**9**	**Sieg–Irsenbachmündung**	50.7813	7.6274	9	fishes / invertebrates
10	Agger–Troisdorf B8	50.8126	7.1876	10	fishes
11	Agger–Wahlscheid	50.8915	7.2468	9	fishes
12	Sieg–Altarm Wolsdorf	50.7906	7.2301	-	fishes
13	Sieg–Krabachmündung	50.7621	7.3993	10	fishes
14	Derenbach—nah Mündung	50.7969	7.3422	1	fishes
15	Derenbach	50.8060	7.3679	1	fishes
16	Derenbach	50.8094	7.3982	1	fishes
17	Bröl–unten	50.7848	7.3102	9	fishes
18	Bröl–oben	50.7881	7.3322	9	fishes
19	Krabach–nah Mündung	50.7619	7.3996	2	fishes / invertebrates
20	Krabach	50.7643	7.4109	2	fishes / invertebrates
21	Krabach	50.7567	7.4122	2	fishes / invertebrates
22	Krabach–WRRL-PS P314	50.7336	7.4254	1	fishes
23	Eipbach–oberhalb HRB	50.7233	7.4516	-	fishes
24	Eipbach–unterhalb HRB	50.7300	7.4502	1	fishes
25	Wohmbach	50.7236	7.4536	-	fishes
26	Wohmbach	50.7286	7.4800	-	fishes
27	Wahlbach	50.7906	7.3292	-	invertebrates
28	Wahlbach	50.7930	7.3227	-	invertebrates
29	Wahlbach	50.7957	7.3229	-	invertebrates
30	Wahlbach	50.7968	7.3227	-	invertebrates
31	Wahlbach	50.7972	7.3212	-	invertebrates
32	Wahlbach	50.7980	7.3175	-	invertebrates

The nine main sample locations are marked by bold type.

Juvenile fishes were sampled and then morphologically identified by the applied fisheries biologist Dipl. Biol. Ivar Steinman (www.fischereibiologe.de), who regularly implements the required WFD monitoring and quality assessment for the BQE ‘fishes’ by order of the NRW state government. Sampling was conducted by using electro-fishing (direct current (500 V; 5 A) to minimize possible stress to the fishes), by boat as single passes or by wading using a point-abundance approach. Each stream section sampled comprises a distance > 100m (sampling area by wading: 40 times the stream width; from boat: 100 times–following [36]), considering all microhabitat types present per reach.

Nine main sampling locations (Fig 1) were investigated, supplemented by 23 additional sites, covering together the variety of aquatic habitats in the River Sieg and its tributaries (Table 1). This strategy covered the different fish water types of the state NRW (https://www.flussgebiete.nrw.de/fischgewaessertypen-5585) in the range of FiGt_01 (upper trout type, low mountain range) to FiGt_11 (lower barbel type, low mountain range), supplemented by small tributaries without type classification (Table 1). Due to low individual numbers and species coverage at the nine main sampling points, additionally seine netting was used as alternative method to estimate species composition and abundances in detail; subsamples were randomly taken to determine the time effort needed for identification of juveniles and larvae. Fishes were humanely sedated and euthanized in chlorobutanol (1,1,1-trichloro-2-methyl-2-propanol) conforming to the Directive 2010/63/EU (all the permissions requested under the German law had been granted).

All specimens–juvenile fishes and aquatic invertebrates–collected in this study were then preserved immediately in 95% instead of 70% ethanol used in WFD standard protocols, and are permanently deposited in the ichthyology collection of the Zoologisches Forschungsmuseum Alexander Koenig (ZFMK) in Bonn. The individuals of both organism groups are associated with the German Barcode of Life project. Detailed specimen data (taxonomy, collection sites, and voucher catalogue numbers) and sequences are available on BOLD (Barcode of Life Data System) under doi.org/10.5883/DS-GBOLFISH and doi.org/10.5883/DS-GBOLMZB or www.bolgermany.de.

### Specimen identification and processing

For assessing general success in taxa detection and taxonomic resolution of both identification approaches in detail the general steps of the standard monitoring routines for WFD in NRW, Germany were followed. In the first step after sample collection and counting, fish and invertebrate specimens were sorted, separated and then morphologically identified by the respective WFD experts (Dipl. Biol. Ivar Steinman / Dr. Guido Haas)—using if necessary, a microscope—to the required or possible taxonomic level. In fishes this is species-level [36] and in macroinvertebrates at least the level required by the national operational German taxa list, containing additionally information about which determination keys should be used per taxa [37]. For fishes, beside the taxonomic community composition and species abundances, the age structure was determined.

In the second step, DNA barcoding routines with bidirectional Sanger-sequencing of the same fish and macroinvertebrate individuals morphologically processed in detail by WFD experts were performed. To this end, a single leg, a tissue sample or a fin clip were taken from each individual, sorted into 96-well plates, and prepared for DNA sequencing. This followed standard DNA barcoding routines at ZFMK, with DNA extraction, PCR amplification and sequencing (described in detail e.g. by [38] for fishes, and [39] for aquatic invertebrates).

During each step of sample processing to the endpoint where further analyses can be made, associated costs and time were estimated, including final error checking, and second round sequencing (if necessary).

### Analysis

Based on the aquatic macroinvertebrate taxa lists (including all individuals processed by ‘live-sorting’ on site) the water quality classification follows the standards of PERLODES, the German river classification system within the ASTERICS (AQEM/STAR Ecological River Classification System) software version 4.0.4 (www.fliessgewaesserbewertung.de). Beside the standardised WFD quality assessment, in this study the River Sieg was additionally classified by the individual expert knowledge. The German fish-based evaluation system, FiBS (www.flussgebiete.nrw.de) version 8.0.6 was used to assess the ecological status of the sampling sites by comparing the generated fish taxa lists to the stream-specific fish faunistic references.

Obtained DNA barcodes were first compared to the available sequences on the BOLD reference database (BOLD ID engine). Barcodes that showed a match of ≥99% to the closest library sequence were assigned a species-level identification, ≥95% similarity confirms genus-level, ≥90% family-level, ≥85% order-level; the resulting molecular-based taxonomic assignments were subsequently compared to the prior generated morphology-based identifications. Discrepancies (caused by potential misidentifications or errors in the BOLD database) were marked and used to morphologically re-inspect the affected specimens and, if necessary, to revise the taxonomic identification; the COI-based dataset revision was made in consultation with the respective WFD expert (Dr. Guido Haas / Dipl. Biol. Ivar Steinman) to ensure proper species-level assignments. Finally, the cleaned barcode sets were uploaded to BOLD and automatically assigned to new or existing ‘Barcode Index Numbers (BINs)’ through the Refined Single Linkage (RESL) algorithm [40]. The ‘BIN Discordance Report’ (BOLD v3) exposes potential taxonomic conflicts within a BIN; BINs were classified as concordant, if they contain specimens with only one taxon name of the same rank.

Identification congruences and discrepancies were visualized in neighbour-joining (NJ) trees [41], including the individual BIN assignment. Using the MUSCLE alignment [42] and Kimura 2 parameter distance model, the trees were calculated with BOLD. Exemplary for the macroinvertebrates of the six main sample points, an UpSet Plot [43] was used to show differences in the combinations of intersections in species presence or absence at the six sample points when using both identification methods.

## Results

### Aquatic macroinvertebrates

At the six main sampling points of the River Sieg a total of 9988 individuals from 101 different taxa (including family & genus, species) were directly identified *in situ* by a single experienced consultant. Concordant with the official WFD protocol [34], individuals of six taxa (Ceratopogoninae/ Palpomyiinae Gen. sp. (14768 taxa ID number), Chironomidae Gen. sp. (4642 taxa ID number), Tanypodinae Gen. sp. (6972 taxa ID number), Spongillidae Gen. sp. (8846 taxa ID number), Naididae Gen. sp. (6068 taxa ID number), and Tubificidae Gen. sp. (7117 taxa ID number)) were just identified and quantified in field and thus not target of the barcode analysis. Based on the taxa list generated, the expert and the German assessment system PERLODES (software ASTERICS) classified four of the sample points (Bergheim, Brölmündung, Irsenbach, Schladern) as “good“, and one (Aggermündung) as “moderate“. In one case the expert opinion differs from PERLODES, assessing the water quality of Bülgenauel as “moderate“, instead of “good“. After live-sorting in the field, for the comparative analysis of identification congruence and taxonomic resolution 720 macroinvertebrates (out of 95 taxa–see above) were separated, preserved in >95% ethanol and morphologically identified to the required or possible taxonomic level. This identification took the taxonomy expert about 36 hours (3min per individual) with costs of 2.86€ per specimen on average (Table 2).

**Table 2:** Direct comparison of time and cost effort of both identification methods.

variable	DNA-based				morphology-based	
	identification (aquatic invertebrates/fishes)				Identification	
	DNA extraction*	PCR	Sanger-sequencing ****	data analysis *****	aquatic invertebrates	fishes
		^a^HotStar Taq**, ^b^standard Taq***	^c^bi-directional, ^d^fw. or rv. only			
**no. specimens simultaneously**	96	96	96	96	1	1
**time**	**5 h**	**3.5 h**	**2 h** (waiting 2–10 days)	**2 h**	**3 min**	**9 sec**
**costs**	96*2.50 €	^a^ ca. 96*1.10 €	^c^ca. 96*5.00 €		**2.86€**	**0.15€**
		^b^ ca. 96*0.30 €	^d^ca. 96*2.50 €			

*DNA Extraction—Macherey & Nagel NucleoSpin®

**QIAGEN Multiplex PCR Kit /

***PCR Core Kit

****Sanger-sequencing—Macrogen (South Korea)

*****Data analysis: DNA sequencing—Geneious

Although with DNA barcoding 96 samples were processed simultaneously on one plate it is more costly (5.30–8.60€ vs. 0.15–2.86€ per sample) and time consuming (12.5h without waiting time vs. 0.15-3min).

Subsequently, 638 morphologically identified specimens (for the frequent species *Esolus parallelepipedus* subsamples were taken), covering each of the 95 macroinvertebrate taxa of the six main sampling points, were analysed together with further 221 morphologically identified specimens (from 73 taxa; with 30 different from the six main sample points) from the tributaries Wahlbach and Krabach by DNA barcoding. Thus 859 specimens from 125 taxa (including 91 species, 32 genera & 2 families) were included in the subsequent method comparison.

From the 859 (six sample points: 638 + tributaries: 221) morphologically identified specimens analysed with DNA barcoding, in total 639 DNA barcode sequences– 466 from the six main sample points and 173 of the two tributaries (out of 108 morphologically identified taxa including 84 spec., 23 gen., & 1 fam.)–were generated successfully. This resulted in a general workload of up to 12.5 hours for a 96-well plate (sending plates for the sequencing step to Macrogen results in further 2–10 days waiting for results), with costs of ca. 8.60€ per specimen (Table 2) when using the HotStar Taq-polymerase (QIAGEN Multiplex PCR kit) and bi-directional sequencing; costs lowered, down to 5.30€, when cheaper Taq-polymerase and forward or reverse only were used. The barcode recovery ranged from 100% in amphipods and isopods, to 90.9% in Plecoptera, to 85.4% in Trichoptera, 83.3% Diptera, 79.5% in Ephemeroptera, and 51.1% in Coleoptera, to only 3.8% in plathelminths.

The direct comparison of the previously generated morphological identification vs. the BOLD ID engine (Table 3) revealed in 74.96% of the 639 sequences a 1:1 match at species-level, whereas 7.04% showed dissimilarities in the identification to species-level; the respective 45 specimens of 22 morphology-based taxa were now genetically assigned to 25 COI-based taxa (Table 4A). Out of the 125 specimens identified to genus-level or higher by morphology only, 92% (115 specimens, 18% of the 639) could be assigned to a reference database entry (>99% ID) and thus to a species (Table 4B). In 10 individuals this was only possible to genus-level, presenting no change compared to the morphological identification (thus included in the 74.96% 1:1 match above).

**Table 3:** Comparison of the morphological identification vs. the identification through the BOLD ID engine.

							method comparison		
				# individuals prior morphologically identified at			morphological identification vs. BOLD ID engine		
	# specimens sampled	# specimens barcoded	# sequences generated	species-level	genus-level	family-level	1:1 match	mismatch	assigned to species
**six main sample points River Sieg**	720	638	466	381	84	1	363	27	76
**two tributaries (Wahlbach/Krabach)**	221	221	173	133	39	1	116	18	39
**# total**		859	**639**	514	123	2	**479**	**45**	**115**

From the 859 morphologically identified specimens processed by DNA barcoding, sequences were generated for 639. For these specimens the prior identification level is given as well as the results of method comparison.

**Table 4:** Dissimilarities in the identification–results of the taxonomical dataset revision. **a)** 45 specimens of 22 prior morphologically identified taxa were assigned to 25 taxa (species-level) by DNA barcoding (>99% ID); **b)** the barcodes of 115 specimens identified by the expert to genus-level or higher could be assigned to a reference database entry (>99% ID) and thus to a species.

a)			b)		
prior expert identification	COI-based	# specimens	prior expert identification	COI-based	# specimens
*Athripsodes cf. *bilineatus*	*Athripsodes albifrons*	**4**	*Baetis* sp.	*Baetis fuscatus*	**1**
*Baetis cf. *vardarensis*	*Baetis lutheri*	**1**	*Baetis* sp.	*Baetis vernus*	**3**
*Centroptilum cf. *luteolum*	*Baetis niger*	**1**	*Chaetopterygini Gen*. sp.	*Chaetopteryx villosa*	**1**
*Caenis cf. *luctuosa*	*Caenis macrura*	**1**	*Dicranota* sp.	*Dicranota bimaculata*	**2**
*Micropterna cf. *lateralis/sequax*	*Chaetopteryx major*	**1**	*Dicranota* sp.	*Dicranota gracilipes*	**2**
*Ecdyonurus cf. *insignis*	*Ecdyonurus dispar*	**1**	*Dicranota* sp.	*Dicranota pavida*	**1**
*Ecdyonurus cf. *torrentis*	*Ecdyonurus dispar*	**1**	*Ecdyonurus* sp.	*Ecdyonurus dispar*	**3**
*Ecdyonurus cf. *torrentis*	*Ecdyonurus insignis*	**1**	*Ecdyonurus* sp.	*Ecdyonurus insignis*	**5**
*Ecdyonurus cf. *insignis*	***Ecdyonurus subalpinus	**1**	*Ecdyonurus* sp.	***Ecdyonurus subalpinus	**2**
*Elmis cf. *aenea*	*Elmis maugeti*	**2**	*Elmis* sp.	*Elmis maugeti*	**10**
*Elodes cf. *minuta-Group*	*Elodes marginata*	**1**	*Esolus* sp.	*Esolus parallelepipedus*	**4**
*Erpobdella cf. *octoculata*	*Erpobdella nigricollis*	**3**	*Esolus sp.	*Oulimnius tuberculatus*	**1**
*Gammarus cf. *pulex*	*Gammarus fossarum*	**2**	*Gammarus* sp.	*Gammarus fossarum*	**4**
*Habroleptoides cf. *confusa*	*Habrophlebia lauta*	**1**	*Hydraena* sp.	*Hydraena gracilis*	**5**
*Halesus digitatus/tesselatus	*Halesus digitatus*	**2**	*Hydroptila* sp.	*Hydroptila simulans*	**3**
*Hydropsyche pellucidula-Group	*Hydropsyche incognita*	**3**	*Hydroptila* sp.	*Hydroptila sparsa*	**3**
*Leuctra cf. *geniculata*	*Leuctra fusca*	**7**	*Isoperla* sp.	*Isoperla goertzi*	**12**
*Limnius cf. *volckmari*	*Limnius opacus*	**1**	*Leuctra* sp.	*Leuctra fusca*	**6**
*Limnius cf. *perrisi*	*Limnius volckmari*	**1**	*Limnius* sp.	*Limnius opacus*	**10**
*Plectrocnemia cf. *conspersa*	*Polycentropus flavomaculatus*	**1**	*Limnius* sp.	*Limnius volckmari*	**3**
**Plectrocnemia cf. *conspersa*	*Potamophylax cingulatus*	**1**	*Nemoura* sp.	*Nemoura marginata*	**1**
*Asellus cf. *aquaticus*	*Proasellus coxalis*	**2**	*Niphargus sp.	*Gammarus pulex*	**1**
*Centroptilum cf. *luteolum*	*Procloeon bifidum*	**1**	*Pedicia* sp.	*Pedicia littoralis*	**2**
*Sericostoma schneideri*	***Sericostoma baeticum	**1**	*Prosimulium sp.	*Simulium lineatum*	**1**
*Simulium cf. *equinum*	*Simulium lineatum*	**4**	*Prosimulium sp.	*Simulium reptans*	**1**
			**Protonemura sp.	*Isoperla goertzi*	**1**
			*Protonemura* sp.	*Protonemura auberti*	**3**
			*Radix* sp.	*Radix balthica*	**1**
			*Rhyacophila* sp.	*Rhyacophila nubila*	**13**
			*Sericostoma* sp.	*Sericostoma baeticum*	**6**
			*Simulium* sp.	*Simulium lineatum*	**2**
			*Simulium* sp.	*Simulium reptans*	**1**
			*Tanytarsini*	*Tanytarsus heusdensis*	**1**

* immature/early larval stage /

**incorrect tube label assignment/

*** barcode-based assignment needs further investigation

Taken together, the 639 DNA barcode sequences were finally assigned in total to 104 different taxa, including 100 species and 4 genera. 21 species were just detected by barcoding, 7 prior morphologically identified species could not be confirmed by the DNA-based identification approach.

**Fig 2:**
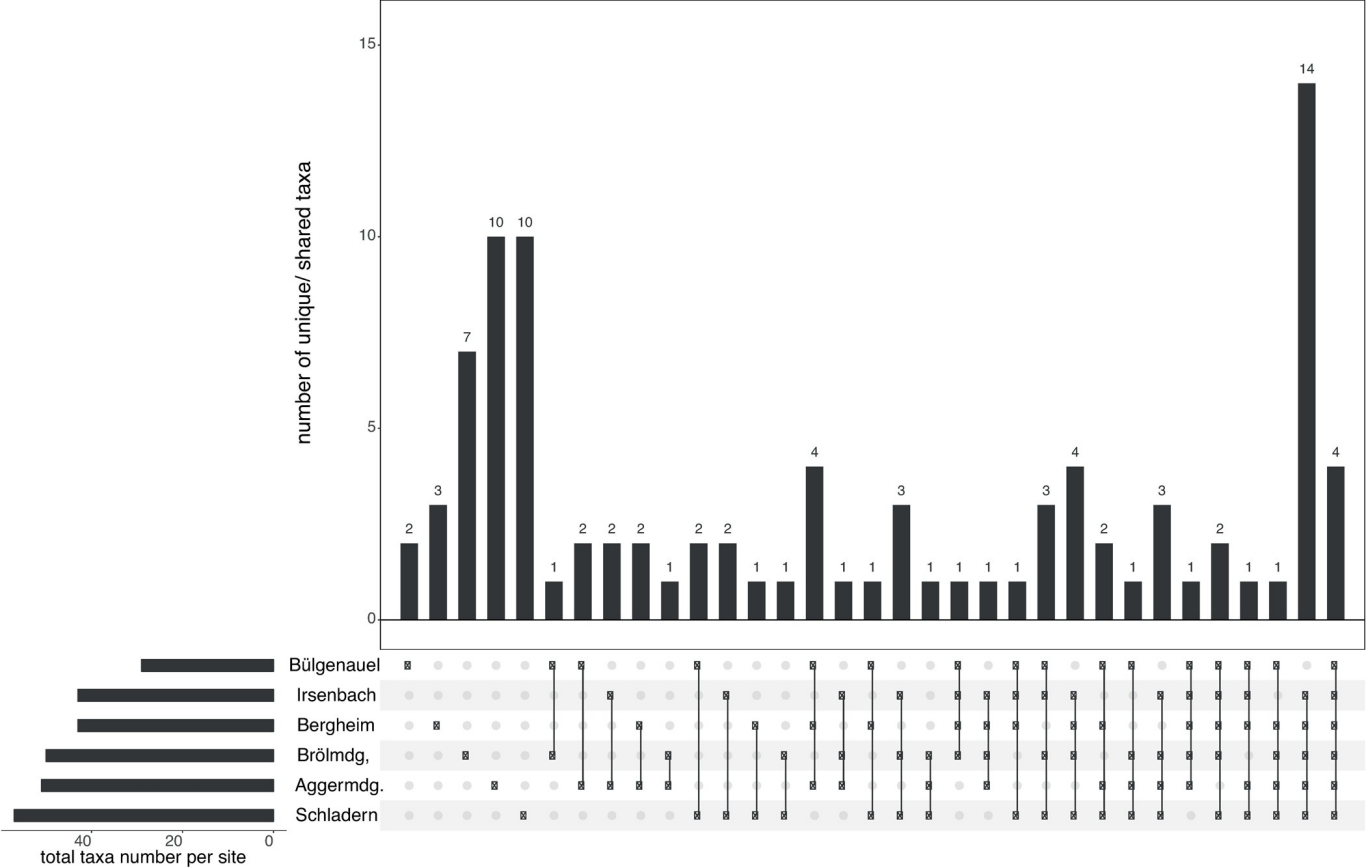
UpSet plot showing the distribution pattern of macroinvertebrate taxa identified by morphology. UpSet plot showing the distribution of the 95 macroinvertebrate taxa (including 71 species and 24 with coarser taxonomy) determined by morphological identification across the six sample points (main stream)–e.g. 2 taxa were found only at Bülgenauel (left), whereas 4 taxa were present at each sample point (right).

**Fig 3:**
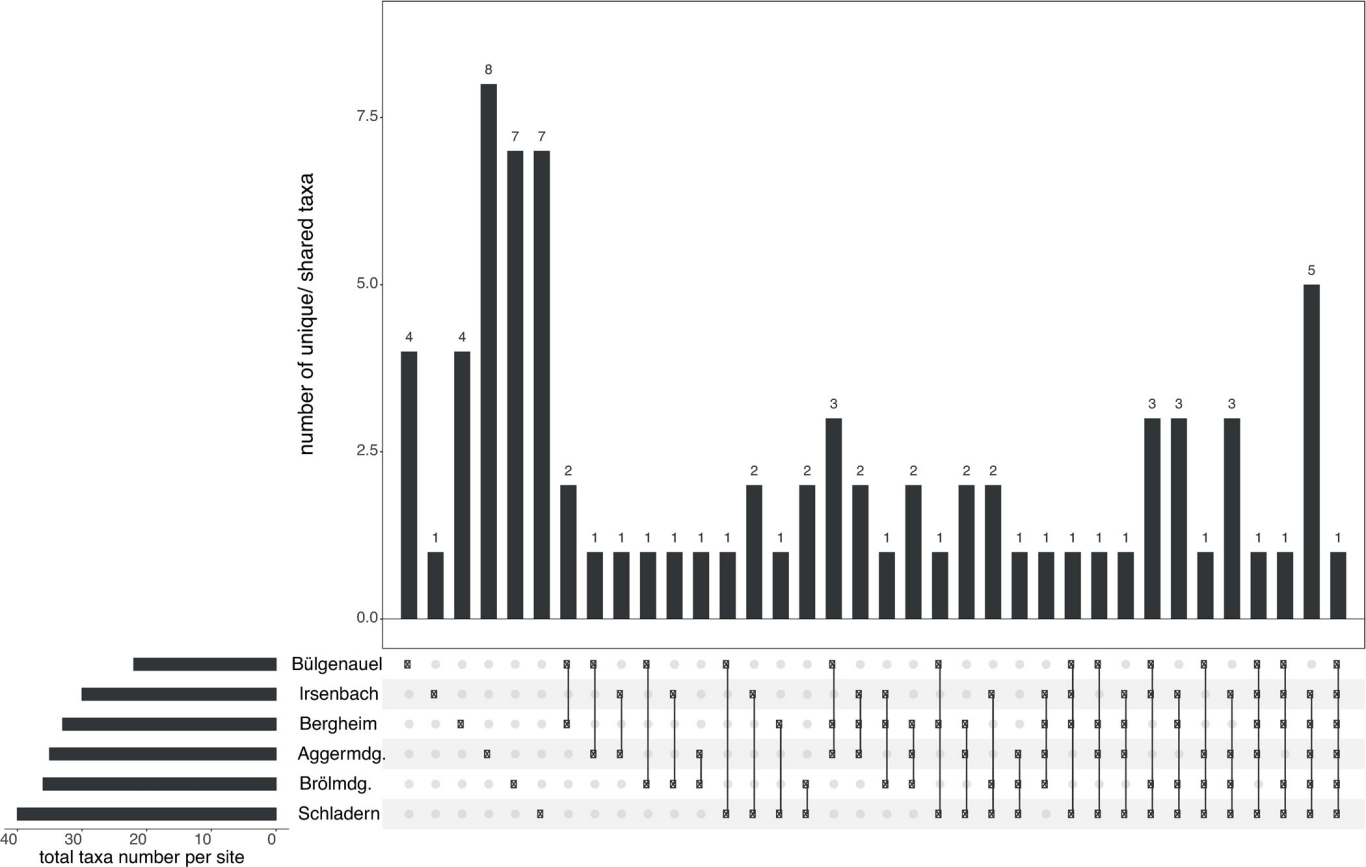
UpSet plot showing the distribution pattern of macroinvertebrate taxa identified by DNA barcoding. UpSet plot showing the distribution of 80 taxa (including 77 species and 3 genera) across the six different sample points (main stream) of macroinvertebrates based on identification through DNA barcoding–e.g. 4 taxa were found only at Bülgenauel (left), whereas one taxon was present at each sample point (right).

The UpSet plots (showing dataset intersections) were used to visualise how the unique/ shared taxa numbers and their distribution patterns across the six main sample points change with the application of the two different identification approaches. With the classical morphology-based identification approach, out of 95 *in situ* identified taxa (including 1 family, 23 genera and 71 species) only two were just present at Bülgenauel, three at Bergheim, seven at Brölmündung, and ten at Aggermündung and Schladern (Fig 2 and Table 5), whereas four species were found at each sampling point (Fig 2 and Table 5; remaining taxa distribution patterns are presented in S1 Table). In contrast, DNA barcoding identified 80 taxa (including 77 species and 3 genera) with four species found to be present only at Bülgenauel, one at Irsenbach, four at Bergheim, eight at Aggermündung, seven at Brölmündung, and seven at Schladern (Fig 3 and Table 5); just one species (*Serratella ignita*) was found at each sample point (Fig 3 and Table 5; remaining taxa distribution patterns are presented in S1 Table). Highest diversity was found with 57 morphology-based and 40 COI-based taxa (41 BINs) at Schladern (Fig 3 and Table 5).

**Table 5:** Direct comparison of taxa found at the six main sample points when using the two different identification approaches.

	morphology-based		COI-based	
sample point	taxa present at only one sample point	total taxa number found*	taxa present at only one sample point	total taxa number found
**Bülgenauel**	*Anabolia nervosa*, *Nebrioporus depressus*	29	*Caenis luctuosa*, *Anabolia nervosa*, *Proasellus coxalis*, *Nebrioporus depressus*	22
**Irsenbachmündung**	no one	43	*Simulium equinum*	30
**Bergheim**	*Caenis rivulorum*, *Alainites (Baetis) muticus*, *Athripsodes* sp.	43	*Caenis rivulorum*, *Pisidium* sp., *Alainites (Baetis) muticus*, *Tanytarsus heusdensis*	33
**Aggermündung**	*Athripsodes albifrons*, *Baetis sp*., *Polycelis* sp., *Anomalopterygella chauviniana*, *Onychogomphus forcipatus*, *Oecetis notate*, *Simulium lineatum*, *Dina lineata*, *Calopteryx virgo*, *Goera pilosa*	51	*Athripsodes albifrons*, *Onychogomphus forcipatus*, *Baetis niger*, *Anomalopterygella chauviniana*, *Oecetis notate*, *Dina lineata*, *Calopteryx virgo*, *Goera pilosa*	36
**Brölmündung**	*Micrasema longulum*, *Heptagenia suphurea*, *Lasiocephala basalis*, *Halesus digitatus/tesselatus*, *Agraylea multipunctata*, *Chaetopteryx villosa*, *Potamopyrgus antipodarum*	50	*Micrasema longulum*, *Halesus digitatus*, *Heptagenia suphurea*, *Chaetopteryx villosa*, *Dugesia gonocephala*, *Potamopyrgus antipodarum*, *Hydropsyche pellucidula*	36
**Schladern**	*Elmis rioloides*, *Radix* sp., *Habrophlebia confusa*, *Ceraclea albimacula*, *Glossiphonia complanata*, *Plectrocnemia conspersa*, *Helobdella stagnalis*, *Orectochilus villosus*, *Antocha* sp., *Leuctra fusca-*Group	57	*Habrophlebia lauta*, *Ceraclea albimacula*, *Glossiphonia complanata*, *Helobdella stagnalis*, *Orectochilus villosus*, *Caenis macrura*, *Radix balthica*	40
**present at each sample point**	*Serratella ignita*, *Aphelocheirus aestivalis*, *Simulium* sp., *Oulimnius tuberculatus*		*Serratella ignita*	

Note: *morphology- base **total taxa numbers** are higher because they contain beside misidentifications specimens which were assigned to different taxa or taxonomic levels although belonging to one species; par example, specimens which were listed by the expert in 3 different taxa: *Elmis maugetii*, *Elmis aenea*, and *Elmis* sp., all belong based on COI data to *Elmis maugeti* (BOLD sequence match of >99%).

Direct comparison of taxa found at only one and species present at each of the six main sample points based on classical morphology-based identification (including family & genus, species) vs. DNA barcoding. Further, the total number of taxa found at each sample point is given.

After taxonomical dataset revision, the 639 sequences were finally clustered by BOLD into 113 BINs, including five new to BOLD (as of date Nov 2, 2018) (*Baetis vardarensis* BOLD:ADM7406; *Pisidium* sp. BOLD:ADM7550; *Sphaerium corneum* BOLD:ADM7571; *Serratella ignita* BOLD:ADM8860; *Dina lineata* BOLD:ADO1748). Individuals of seven previously identified taxa split each into two BINs (*Asellus aquaticus* BOLD:AAA1970, BOLD:ACF1266; *Atherix ibis* BOLD:ACG1351, BOLD:ACO4109; *Baetis rhodani* BOLD:AAE4621, BOLD:AAM1760; *Eiseniella tetraedra* BOLD:AAB7509, BOLD:AAB7510; *Gammarus pulex* BOLD:ADD3272, BOLD:ADD3276; *Limnius opacus* BOLD:AAF4988, BOLD:ACZ1035; *Niphargus* sp. BOLD:ACQ7274, BOLD:ADM7126), whereas specimens of *Serratella ignita* cluster into three (BOLD:AAB3693, BOLD:AAZ7536, BOLD:ACB0418).

The BIN discordance report (Nov 21, 2018) revealed that 55.75% of the 113 BINs were found to be concordant, one was represented by a single individual, and 29 among all BINs were discordant (see NJ tree: S1 Fig). The NJ tree shows discrepancies/ conflicts in identification accuracy and taxonomic resolution between both identification methods, BIN numbers are included (S1 Fig).

### Freshwater fishes

**Fig 4:**
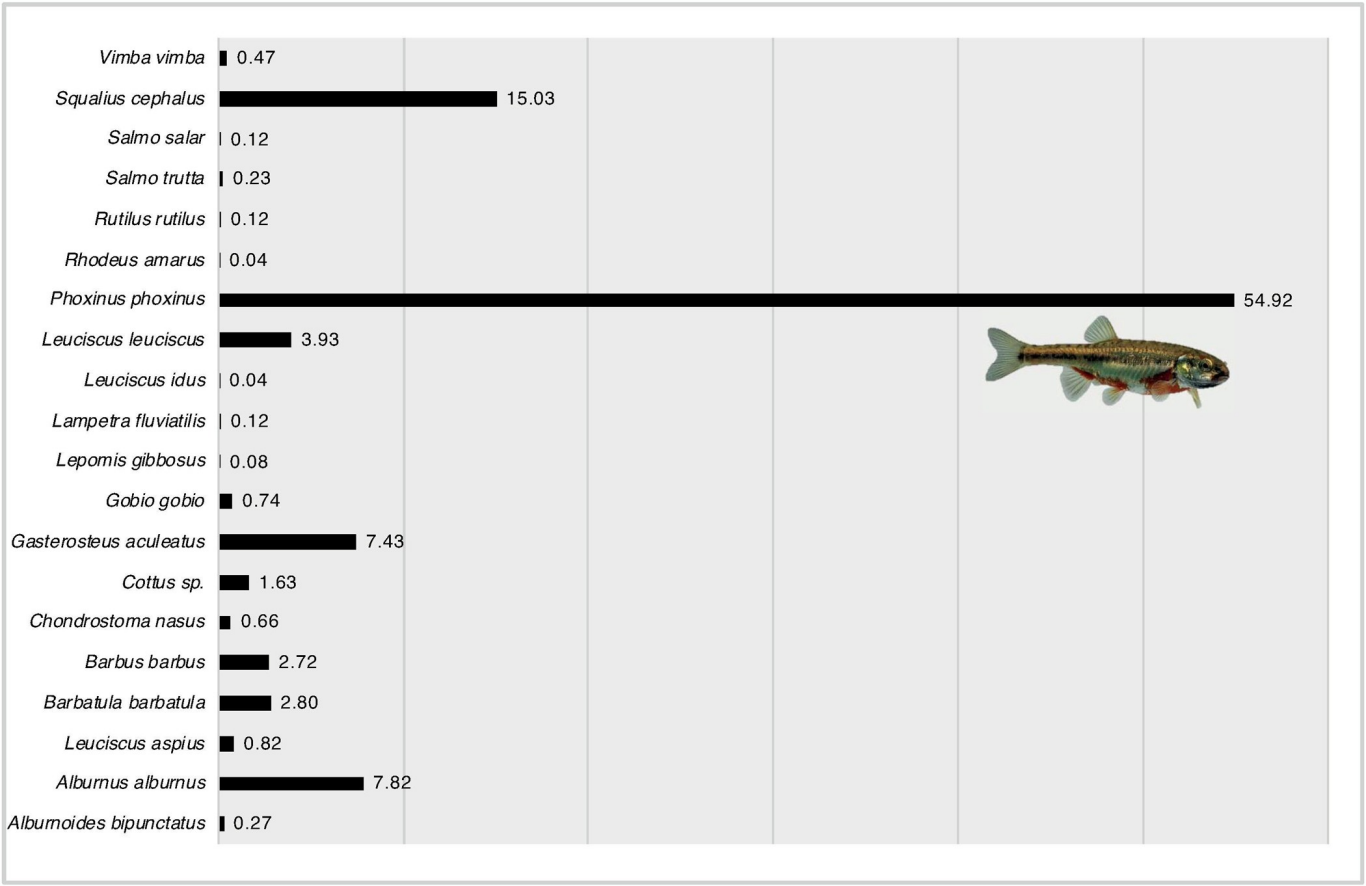
Composition of 20 species (2569 individuals) in juvenile fishes (+0, first year of age) in %.

Standard WFD electro-fishing in the years 2012 and 2013 revealed 2569 juvenile fishes (0+) that were subsequently identified by one expert. 20 fish species were detected based on morphological characters (Fig 4).

A total of 715 DNA barcode sequences were successfully generated for the juvenile (0+) fish sample, whereas 134 specimens (including all juvenile *S*. *salar* and *S*. *trutta*) failed to produce a DNA barcode (84.2% success rate). Sample processing time and costs for barcoding routines remain the same as in aquatic invertebrates (Table 2). The direct comparison of both identification methods using the BOLD ID engine yielded a 1:1 match in 99.03% of the specimens. Only 0.97% (seven 0+ specimens) showed a discrepancy between the morphological identification and the COI data (see NJ tree: S2 Fig). The 715 sequences of 18 species were assigned to 20 BINs, shown in the NJ tree (S2 Fig). Individuals of *B*. *barbatula* were split into two (BOLD:AAA1238, n = 37; BOLD:AAA1239, n = 1), *P*. *phoxinus* into three (BOLD:AAC8036, n = 82; BOLD:AAY8765, n = 7; BOLD:ACE5740, n = 81) different BINs. According to the BIN discordance report (Nov 21, 2018), 25% were assigned to concordant BINs and 15 BINs were found to be discordant (see NJ tree: S2 Fig).

Probably due to a long winter in 2012/13 the reproductive success of the fish community in the River Sieg was severely restricted in that year. Therefore, the numbers and species coverage (only 40% of the 50 species listed in [33]) in the juvenile fish were significantly below expectations; fish larvae and eggs were missing entirely. The water quality of five sample points (Irsenbachmündung, Happach, Bülgenauel, Röcklingen, Schladern) was assessed by using FiBS as “poor”, whereas three locations (Pleisbachmündung, Aggermündung, Bergheim) were classified as “bad” and only one (Brölmündung) as “moderate”.

It took the expert 7 hr and 47 min to identify 36 randomly chosen seine net fishing subsamples of 3164 individuals in total, resulting in 6.78 individuals per minute with a rough cost of 0.15€ per sample (Table 2).

## Discussion

This application study aimed at evaluating potential advantages in taxa detection and taxonomic resolution when DNA barcoding supplements the identification process of stream monitoring routines. In most standard water bioassessments, many organisms are determined to higher levels such as genera or family only, in order to minimize processing time- and hence maximize cost efficiency [44,45]. As in some aquatic invertebrate taxa even closely related species can vary substantially in their ecological tolerance and respond different to environmental disturbances, the consequence of this traditional approach is a potential information loss, which may moreover result in inaccurate water quality evaluations [13,19,24].

The present results underline, consistent to e.g. Sweeney et al. [13], Stein et al. [24] and Elbrecht et al. [27] that sequence-based bioassessments can capture biodiversity with increased taxonomic resolution and precision, resulting in a more complete community structure description with the opportunity to document and quantify even small changes in freshwater ecosystems. Especially in aquatic invertebrates, the direct method comparison showed that DNA barcoding produces a more detailed taxa list with species which were not detected based on morphological traits while further formerly identified species could not be confirmed.

When comparing the overall taxa numbers of the taxonomic inventories in aquatic invertebrates, further discrepancies in accuracy between both identification approaches get obvious. With the use of coarser-scale taxonomy, the expert listed a higher taxa amount (101 vs. 80 at the six sample points of main stream) because beside misidentifications, morphological challenging specimens actually belonging to one species were assigned to different taxa or taxonomic levels (see note Table 5). Here, the incorporation of DNA barcodes provided more accurate and objective species-level data, clearly changing the detection of taxa occurrence and their abundance patterns per sample point (Figs 2 and 3 and Tables 5 and S1).

Through enhancing taxonomic resolution and including individuals of each size, sex, life stages and/ or even damaged samples in environmental quality analyses, DNA barcoding allowed to gain a more complete reflection of the ecological community present [13,28,46,47]. By putting the barcodes into context with the reference sequences data on BOLD through BIN assignment [40], genetic variation was found, which requires further detailed studies (e.g. in *Barbatula*, *Limnius*, *Serratella*); in the minnows of the River Sieg the three distict haplotypes can be assigned to P. *phoxinus* (BOLD:ACE5740), *P*. *csikii* (BOLD:AAC8036) and *P*. *septimaniae* (BOLD:AAY8765, based on Palandačić et al. [48,49]. In general, the assignment to multiple BINs indicates the presence of regional genetic variants or even cryptic, unrecognized species [19,50–52]. Both might theoretically harbor genetic diversity, which leads to variation in adaptation to local environmental conditions and thus (if of autochthonous origin) providing ecological information with importance for freshwater resource protection and conservation planning [18,19,53].

Species-level identifications generated by DNA-based methods highly depend on the coverage and quality of the reference database used [26,54–57]. For example, for the genera *Sericostoma* (Trichoptera), *Niphargus* (Amphipoda), *Pisidium* (Mollusca) and the tribe *Tanytarsini* (Diptera) the current barcode library contains not sufficient reference data to generate species-level identifications (see NJ tree: S1 Fig). Extension of reference data through single specimen DNA barcoding based on properly determined individuals stored in reference collections is required for filling these gaps. With the present study 5 new BIN entries for aquatic invertebrates could be added to the BOLD library, representing previously missing genetic entities.

Apart from the identification and closing of existing data gaps to create a more complete reference database, additional effort is needed to resolve taxonomic errors in BOLD, in order to enhance the identification success and robustness also for DNA-based biomonitoring [54,57]. We found specimens assigned by BOLD to species (*E*. *subalpinus*, *S*. *baeticum*) whose occurrence in the River Sieg and tributaries are rather excluded [58,59]. Additonally, 48% of all BINs to which barcodes of this study were assigned contain between 2 and 19 different names. Such taxonomic inconsistencies or errors present in the global library for freshwater organisms at family-, genus- or species-level may result from artefacts like inadequate prior taxonomic assignment, synonymies, or inadequate data management with the lack of taxonomical updates in the database [54,60]. Here, the comprehensive knowledge of well-trained taxonomists is needed to further increase the number of unequivocal species-level assignments using DNA barcodes [60]. The diagnostic utility of COI barcodes can also be restricted by haplotype sharing through natural processes like hybridization, introgression or incomplete lineage sorting in young species [29,30,61]. The combination of mitochondrial and nuclear markers may help to overcome such uncertainties [62–64].

Despite refining taxonomic resolution, our detailed time-cost analysis of both methods additionally showed, similar to Stein et al. [65], that single specimen DNA barcoding based on Sanger-sequencing is at this developmental stage still too expensive and time consuming. Despite the possibility to lower lab costs of conventional Sanger-based barcoding by using par example cheap Taq polymerase and PCR procedures it is not a practicable method for large scale bioassessments, dealing with thousands of individuals [65].

However, the generation of public voucher-based reference barcodes by single specimen barcoding is the foundation for currently emerging future applications like DNA metabarcoding with high-throughput sequencing [25,27,66]. These technical advanced barcoding methods help to save time and money during data acquisition by allowing to process multiple organism groups in parallel from environmental DNA (eDNA) or bulk samples [27,28,67]. Among the remaining challenges for integrating DNA metabarcoding in freshwater monitoring, proper solutions for the still problematic estimation of abundances are to be found, known to be mainly caused by primer bias and a positive correlation of taxon biomass to number of reads [68–70]. The potential of DNA-based bioassessments will be additionally improved by adapted or new established molecular metrices/ indices, not automatically relegating the species or genus-level identification of morphologically inconspicuous taxa [28,67].

## Conclusion

Taken together, the present study underlines that DNA barcoding-based aquatic biomonitoring provides highly reliable data at species-level which improves the understanding of species community composition and hence the assessment results used to make environmental management decisions. The challenge is now, to bridge the gap between science and application routines, by enabling a dialogue between stakeholders involved in current WFD quality assessments and monitoring routines and researchers applying the more or less new DNA-based identification methods [26,60]. Here new projects, like DNAqua-Net [26,60] are mandatory, aiming to cross-disciplinary organize a standardization of specific field and laboratory protocols to ensure consistency and comparability in produced DNA assessment data [27,60].

## Supporting information

S1 Table Supplement to Table 5. Direct comparison of taxa distribution patterns across the six different main sample points based on classical morphology-based identification (including family & genus, species) vs. DNA barcoding.
(XLSX)

S1 Fig NJ tree of the aquatic invertebrate specimens. NJ tree showing the 45 aquatic invertebrate specimens where the COI identification differs from the prior morphological identification and the 115 specimens which are assigned to species-level by barcoding–the 29 discordant BINs are marked (BOLD v3, Nov 21, 2018).
(EPS)

S2 Fig NJ tree of the fish specimens. NJ tree showing the seven fish specimens where COI identification differs from morphological identification–the 15 discordant BINs are marked (BOLD v3, Nov 21, 2018).
(EPS)

## References

[1] Dudgeon D, Arthrington AH, Gessner MO, Kawabata ZI, Knowler DJ, Lévêque C, et al Freshwater biodiversity: importance, threats, status and conservation challenges. Biol Rev. 2006;81: 163–182. 10.1017/S1464793105006950 16336747

[2] Woodward G, Perkins DM, Brown LE. Climate change and freshwater ecosystems: impacts across multiple levels of organization. Philos Trans R Soc Lond B Biol Sci. 2010;365: 2093–2106. 10.1098/rstb.2010.0055 20513717 PMC2880135

[3] Poff NL, Olden JD, Strayer DL. Climate change and freshwater fauna extinction risk In: Hannah L., editor. Saving a Million Species. Island Press/Center for Resource Economics; 2012 pp. 309–336.

[4] CBD (Convention on Biological Diversity). The convention on biological diversity, text and annexes. Montreal: Secretariat of the Convention on Biological Diversity; 1992 Availabe from: https://www.cbd.int/doc/legal/cbd-en.pdf

[5] Buss DF, Carlisle DM, Chon TS, Culp J, Harding JS, Keizer-Vlek HE, et al Stream biomonitoring using macroinvertebrates around the globe: a comparison of large-scale programs. Environ Monit Assess. 2015;187: 4132 10.1007/s10661-014-4132-8 25487459

[6] EU Water Framework Directive (WFD). Directive 2000/60/EC of the European Parliament and of the Council of 23 October 2000 establishing a framework for Community action in the field of water policy. OJ L. 2000;327: 1–72.

[7] Borja A, Bricker SB, Dauer DM, Demetriades NT, Ferreira JG, Forbes AT, et al Overview of integrative tools and methods in assessing ecological integrity in estuarine and coastal systems worldwide. Mar Poll Bull. 2008;56: 1519–1537. 10.1016/j.marpolbul.2008.07.005 18715596

[8] Birk S, Bonne W, Borja A, Brucet S, Courrat A, Poikane S, et al Three hundred ways to assess Europe’s surface waters: an almost complete overview of biological methods to implement the Water Framework Directive. Ecol Indic. 2012;18: 31–41.

[9] Hopkins GW, Freckleton RP. Declines in the numbers of amateur and professional taxonomists: implications for conservation. Anim Conserv. 2002;5: 245–249.

[10] Frobel K, Schlumprecht H. Erosion der Artenkenner, Abschlussbericht im Auftrag des BUND Naturschutz in Bayern eV, Nürnberg. Naturschutz und Landschaftsplanung: Zeitschrift für angewandte Ökologie. 2014;48: 105–113.

[11] Haase P, Murray-Bligh J, Lohse S, Pauls S, Sundermann A, Gunn R, et al Assessing the impact of errors in sorting and identifying macroinvertebrate samples. Hydrobiologia. 2006;566: 505–521.

[12] Haase P, Pauls SU, Schindehütte K, Sundermann A. First audit of macroinvertebrate samples from an EU Water Framework Directive monitoring program: human error greatly lowers precision of assessment results. J North Am Benthol Soc. 2010;29: 1279–1291.

[13] Sweeney BW, Battle JM, Jackson JK, Dapkey T. Can DNA barcodes of stream macroinvertebrates improve descriptions of community structure and water quality?. J North Am Benthol Soc. 2011;30: 195–216.

[14] Marshall JC, Steward AL, Harch BD. Taxonomic resolution and quantification of freshwater macroinvertebrate samples from an Australian dryland river: the benefits and costs of using species abundance data. Hydrobiologia. 2006;572: 171–194.

[15] Pfrender ME, Hawkins CP, Bagley M, Courtney GW, Creutzburg BR, Epler JH, et al Assessing macroinvertebrate biodiversity in freshwater ecosystems: advances and challenges in DNA-based approaches. Q Rev Biol. 2010;85: 319–340. 10.1086/655118 20919633

[16] Ko HL, Wang YT, Chiu TS, Lee MA, Leu MY, Chang KZ, et al Evaluating the accuracy of morphological identification of larval fishes by applying DNA barcoding. PLoS One. 2013;8: e53451 10.1371/journal.pone.0053451 23382845 PMC3561387

[17] Jackson JK, Battle JM, White BP, Pilgrim EM, Stein ED, Miller PE, et al Cryptic biodiversity in streams: a comparison of macroinvertebrate communities based on morphological and DNA barcode identifications. Freshw Sci. 2014;33: 312–324.

[18] Cook B, Page T, Hughes J. Importance of cryptic species for identifying ‘representative’ units of biodiversity for freshwater conservation. Biol Conserv. 2008;141: 2821–2831.

[19] Macher JN, Salis RK, Blakemore KS, Tollrian R, Matthaei CD, Leese F. Multiple-stressor effects on stream invertebrates: DNA barcoding reveals contrasting responses of cryptic mayfly species. Ecol Indic. 2016;61: 159–169.

[20] Kallimanis AS, Mazaris AD, Tsakanikas D, Dimopoulos P, Pantis JD, Sgardelis SP. Efficient biodiversity monitoring: which taxonomic level to study?. Ecol Indic. 2012;15: 100–104.

[21] Mueller M, Pander J, Geist J. Taxonomic sufficiency in freshwater ecosystems: effects of taxonomic resolution, functional traits, and data transformation. Freshw Sci. 2013;32: 762–778.

[22] Beermann AJ, Elbrecht V, Karnatz S, Ma L, Matthaei CD, Piggott JJ, et al Multiple-stressor effects on stream macroinvertebrate communities: A mesocosm experiment manipulating salinity, fine sediment and flow velocity. Sci Total Environ. 2018;610: 961–71. 10.1016/j.scitotenv.2017.08.084 28830056

[23] Whitfield AK, Elliott M. Fishes as indicators of environmental and ecological changes within estuaries: a review of progress and some suggestions for the future. J Fish Biol. 2002;61: 229–250.

[24] Stein ED, White BP, Mazor RD, Jackson JK, Battle JM, Miller PE, et al Does DNA barcoding improve performance of traditional stream bioassessment metrics? Freshw. Sci. 2013;33: 302–311.

[25] Taberlet P, Coissac E, Pompanon F, Brochmann C, Willerslev E. Towards next-generation biodiversity assessment using DNA metabarcoding. Mol Ecol. 2012;21: 2045–2050. 10.1111/j.1365-294X.2012.05470.x 22486824

[26] Leese F, Altermatt F. Bouchez A, Ekrem T, Hering D, Meissner K, et al DNAqua-Net: developing new genetic tools for bioassessment and monitoring of aquatic ecosystems in Europe. Res Ideas Outcomes. 2016;2: e11321.

[27] Elbrecht V, Vamos EE, Meissner K, Aroviita J, Leese F. Assessing strengths and weaknesses of DNA metabarcoding-based macroinvertebrate identification for routine stream monitoring. Methods Ecol Evol. 2017;8: 1265–1275.

[28] Hering D, Borja A, Jones JI, Pont D, Boets P, Bouchez A, et al Implementation options for DNA-based identification into ecological status assessment under the European Water Framework Directive. Water Res. 2018;138: 192–205. 10.1016/j.watres.2018.03.003 29602086

[29] Hebert PDN, Cywinska A, Ball SL, DeWaard JR. Biological identifications through DNA barcodes. Proc R Soc B. 2003;270: 313–321. 10.1098/rspb.2002.2218 12614582 PMC1691236

[30] Hebert PDN, Ratnasingham S, deWaard JR. Barcoding animal life: cytochrome c oxidase subunit 1 divergences among closely related species. Proc R Soc B. 2003;270: S96–S99. 10.1098/rsbl.2003.0025 12952648 PMC1698023

[31] Briem E. Gewässerlandschaften der Bundesrepublik Deutschland: morphologische Merkmale der Fließgewässer und ihrer Auen. Hennef: Dt. Vereinigung für Wasserwirtschaft, Abwasser und Abfall, ATV-DVWK-Arbeitsbericht; 2003.

[32] Sommerhäuser M, Pottgiesser T. Die Fließgewässertypen Deutschlands als Beitrag zur Umsetzung der EG-Wasserrahmenrichtlinie. Limnol aktuell. 2005;11: 13–27.

[33] Freyhof J. Strukturierende Faktoren für die Fischgemeinschaft der Sieg. 1st ed Göttingen: Cuvillier Verlag; 1998

[34] Meier C, Haase P, Rolauffs P, Schindehütte K, Schöll F, Sundermann A, et al Methodisches Handbuch Fließgewässerbewertung—Handbuch zur Untersuchung und Bewertung von Fließgewässern auf der Basis des Makrozoobenthos vor dem Hintergrund der EG-Wasserrahmenrichtlinie. 2006 Available from: https://www.gewaesser-bewertung.de/files/meier_handbuch_mzb_2006.pdf

[35] Meier C, Böhmer J, Biss R, Feld C, Haase P, Lorenz A, et al Weiterentwicklung und Anpassung des nationalen Bewertungssystems für Makrozoobenthos an neue internationale Vorgaben. Abschlussbericht im Auftrag des Umweltbundesamtes. 2006 Available from: http://gewaesser-bewertung.de/files/abschlussbericht_20060331.pdf

[36] Dußling U. Handbuch zu fiBS. Offenbach am Main: Schriftenreihe des Verbandes Deutscher Fischereiverwaltungsbeamter und Fischereiwissenschaftler eV; 2009. Available from: https://www.gewaesser-bewertung.de/files/fibs-handbuch_2009.pdf

[37] Haase P, Sundermann A, Schindehütte K. Operationelle Taxaliste als Mindestanforderung an die Bestimmung von Makrozoobenthosproben aus Fließgewässern zur Umsetzung der EU-Wasserrahmenrichtlinie in Deutschland. Essen: University of Duisburg-Essen; 2006 Available from: https://www.gewaesser-bewertung-berechnung.de/index.php/perlodes-online.html

[38] Geiger M, Herder F, Monaghan M, Almada V, Barbieri R, Bariche M, et al Spatial heterogeneity in the Mediterranean Biodiversity Hotspot affects barcoding accuracy of its freshwater fishes. Molecular Ecology Resources. 2014;14: 1210–1221. 10.1111/1755-0998.12257 24690331

[39] Rulik B, Eberle J, von der Mark L, Thormann J, Jung M, Köhler F, et al Using taxonomic consistency with semi-automated data pre-processing for high quality DNA barcodes. Methods Ecol Evol. 2017;8: 1878–1887.

[40] Ratnasingham S, Hebert PDN. A DNA-based registry for all animal species: the Barcode Index Number (BIN) system. PloS One. 2013;8 10.1371/journal.pone.0066213 23861743 PMC3704603

[41] Saitou N, Nei M. The neighbor joining method—a new method for reconstructing phylogenetic trees. Mol Biol Evol. 1987;4: 406–425. 10.1093/oxfordjournals.molbev.a040454 3447015

[42] Edgar RC. MUSCLE: Multiple sequence alignment with high accuracy and high throughput. Nucleic Acids Res. 2004;32: 1792–1797. 10.1093/nar/gkh340 15034147 PMC390337

[43] Lex A, Gehlenborg N, Strobelt H, Vuillemot R, Pfister H. UpSet: Visualization of Intersecting Sets. IEEE T VIS COMPUT GR. 2014;20: 1983–1992. 10.1109/TVCG.2014.2346248 26356912 PMC4720993

[44] Schmidt-Kloiber A, Nijboer RC. The effect of taxonomic resolution on the assessment of ecological water quality classes. Hydrobiologia. 2004;516: 269–283.

[45] Pinna M, Marini G, Rosati I, Neto JM, Patrício J, Marques JC, et al The usefulness of large body-size macroinvertebrates in the rapid ecological assessment of Mediterranean lagoons. Ecol Indic. 2013;29: 48–61.

[46] Ekrem T, Stur E, Hebert PDN. Female do count: documenting Chironomidae (Diptera) species diversity using DNA barcoding. Org Divers Evol. 2010;10: 397–408.

[47] Kirtiklis L, Palińska-Żarska K, Krejszeff S, Kupren K, Żarski D et al Fopp-Bayat,. Comparison of molecular and morphometric analysis in species discrimination of larvae among five cyprinids from the subfamily Leuciscinae: A tool for sustainable conservation of riverine ichthyofauna. Biologia. 2006;71: 1177–1183.

[48] Palandačić A, Naseka A, Ramler D, Ahnelt H. Contrasting morphology with molecular data: an approach to revision of species complexes based on the example of European Phoxinus (Cyprinidae). MBC Evol Biol. 2017;17, 184. 10.1186/s12862-017-1032-x PMC5549366 28793855

[49] Palandačić A, Kruckenhauser L, Ahnelt H, Mikschi E. European minnows through time: museum collections aid genetic assessment of species introductions in freshwater fishes (Cyprinidae: Phoxinus species complex). Heredity. 2020;124: 410–422. 10.1038/s41437-019-0292-1 31896822 PMC7028953

[50] Hebert PDN, Penton EH, Burns JM, Janzen DH, Hallwachs W. Ten species in one: DNA barcoding reveals cryptic species in the neotropical skipper butterfly Astraptes fulgerator. Proc Natl Acad Sci USA. 2004;101: 14812–14817. 10.1073/pnas.0406166101 15465915 PMC522015

[51] Weiss M, Macher JN, Seefeldt MA, Leese F. Molecular evidence for further overlooked species within the Gammarus fossarum complex (Crustacea: Amphipoda). Hydrobiologia. 2014;721: 165–184.

[52] Weiss M, Weigand H, Weigand AM, Leese F. Genome‐wide single‐nucleotide polymorphism data reveal cryptic species within cryptic freshwater snail species—The case of the Ancylus fluviatilis species complex. Ecol Evol. 2018;8: 1063–1072. 10.1002/ece3.3706 29375779 PMC5773296

[53] Bickford D, Lohman DJ, Sodhi NS, Ng PKL, Meier R, Winker K, et al Cryptic species as a window on diversity and conservation. Trends Ecol Evol. 2007;22: 148–155. 10.1016/j.tree.2006.11.004 17129636

[54] Geiger MF, Moriniere J, Hausmann A, Haszprunar G, Wägele W, Hebert PDN, et al Testing the Global Malaise Trap Program–How well does the current barcode reference library identify flying insects in Germany?. Biodiv Data J. 2016;4. 10.3897/BDJ.4.e10671 PMC5136679 27932930

[55] Morinière J, Hendrich L, Balke M, Beermann AJ, König T, Hess M, et al A DNA barcode library for Germany′ s mayflies, stoneflies and caddisflies (Ephemeroptera, Plecoptera and Trichoptera). Mol Ecol Resour. 2017;17: 1293–1307. 10.1111/1755-0998.12683 28449274

[56] Morinière J Balke M, Doczkal D, Geiger MF, Hardulak LA, Haszprunar G, et al A DNA barcode library for 5,200 German flies and midges (Insecta: Diptera) and its implications for metabarcoding‐based biomonitoring. Mol Ecol Resour. 2019;19: 900–928. 10.1111/1755-0998.13022 30977972 PMC6851627

[57] Weigand H, Beermann AJ, Čiampor F, Costa FO, Csabai Z, Duarte S, et al DNA barcode reference libraries for the monitoring of aquatic biota in Europe: Gap-analysis and recommendations for future work. bioRxiv. 2019;576553 10.1016/j.scitotenv.2019.04.247 31077928

[58] Eiseler B. Bildbestimmungsschlüssel für die Eintagsfliegenlarven der deutschen Mittelgebirge und des Tieflands. Lauterbornia. 2005;53: 1–112.

[59] Neu PJ, Malicky H, Graf W, Schmidt-Kloiber A. Distribution Atlas of European Trichoptera. ConchBooks; 2018.

[60] Leese F, Bouchez A, Abarenkov K, Altermatt F, Borja A, Bruce K, et al Why we need sustainable networks bridging countries, disciplines, cultures and generations for aquatic biomonitoring 2.0: A perspective derived from the DNAqua-Net COST action In: Bohan D., Dumbrell A., Woodward G., Jackson M., editors. Next Generation Biomonitoring. Academic Press; 2018;63–99.

[61] Moritz C, Cicero C. DNA barcoding: Promise and pitfalls. PLoS Biol. 2004;2: e354 10.1371/journal.pbio.0020354 15486587 PMC519004

[62] Monaghan MT, Balke M, Gregory TR, Vogler AP. DNA-based species delineation in tropical beetles using mitochondrial and nuclear markers. Phil Trans R SocB. 2005;360: 1925–1933. 10.1098/rstb.2005.1724 16214750 PMC1609231

[63] Sonnenberg R, Nolte AW, Tautz D. An evaluation of LSU rDNA D1- D2 sequences for their use in species identification. Front Zool. 2007;4: 6 10.1186/1742-9994-4-6 17306026 PMC1805435

[64] Vuataz L, Sartori M, Wagner A, Monaghan MT. Toward a DNA taxonomy of Alpine Rhithrogena (Ephemeroptera: Heptageniidae) using a mixed Yule-coalescent analysis of mitochondrial and nuclear DNA. PLoS One. 2011;6. 10.1371/journal.pone.0019728 PMC3096624 21611178

[65] Stein ED, Martinez MC, Stiles S, Miller PE, Zakharov EV, et al Is DNA barcoding actually cheaper and faster than traditional morphological methods: results from a survey of freshwater bioassessment efforts in the United States?. PloS One. 2014;9: e95525 10.1371/journal.pone.0095525 24755838 PMC3995707

[66] Hajibabaei M, Shokralla S, Zhou X, Singer G, Baird DJ. Environmental barcoding: A next-generation sequencing approach for biomonitoring applications using river benthos. PLoS One. 2011;6: e17497 10.1371/journal.pone.0017497 21533287 PMC3076369

[67] Pawlowski J, Kelly-Quinn M, Altermatt F, Apothéloz-Perret-Gentil L, Beja P, Boggero A, et al The future of biotic indices in the ecogenomic era: Integrating (e)DNA metabarcoding in biological assessment of aquatic ecosystems. Sci Total Environ. 2018;637: 1295–1310. 10.1016/j.scitotenv.2018.05.002 29801222

[68] Piñol J, Mir G, Gomez-Polo P, Agustí N. Universal and blocking primer mismatches limit the use of high-throughput DNA sequencing for the quantitative metabarcoding of arthropods. Mol Ecol Resour. 2014; 1–12. 10.1111/1755-0998.12196 25454249

[69] Elbrecht V, Leese F. Can DNA-based ecosystem assessments quantify species abundance? Testing primer bias and biomass—sequence relationships with an innovative metabarcoding protocol. PLoS One. 2015;10: e0130324 10.1371/journal.pone.0130324 26154168 PMC4496048

[70] Elbrecht V, Peinert B, Leese F. Sorting things out: Assessing effects of unequal specimen biomass on DNA metabarcoding. Ecol Evol. 2017;7: 6918–6926. 10.1002/ece3.3192 28904771 PMC5587478

